# An overview of Metaverse in healthcare. Potential application in forensic and legal medicine

**DOI:** 10.1007/s12024-025-00938-4

**Published:** 2025-02-14

**Authors:** Davide Ferorelli, Gianmarco Sirago, Francesco Calò, Annachiara Vinci, Paolo Visci, Biagio Solarino, Alessandro Dell’Erba

**Affiliations:** Section of Legal Medicine, University of Bari, piazza Giulio Cesare, 11, 70124 Bari, Italy

**Keywords:** Metaverse, Forensic medicine, Legal medicine, Review, Virtual reality

## Abstract

In this review, we holistically consider healthcare as an ecosystem that includes forensic pathology and legal medicine and probe why these areas have largely ignored the use of Metaverse. This effort is directed at understanding the potential impact of virtual worlds on these areas and ultimately asks if forensic sciences are missing an opportunity or multiple opportunities. We reviewed the scientific literature to identify clinical trials and observational or other study types using PubMed Central, Web of Science, Scopus, and Cochrane, including terms related to the Metaverse and healthcare, with a screen of 1,200 relevant articles. Applications were from future clinical applications, training, treatment, prediction/prevention, and diagnosis, focusing on finding blanks in forensic and legal medicine. The study carried out in-depth research with the Metaverse regarding all areas of medicine. Despite this, significant forensic pathology or legal medicine research has yet to be identified. The gap may implicate a critical oversight in forensic areas, specifically autopsies, crime scene investigation, and field reconstruction of legal cases that could benefit from implementing virtual simulations. In this regard, incorporating virtual environments in these areas can provide new training tools, case scenarios, and even a revision mechanism for cold cases, leading to an essential trend in the evolution of legal medicine.

## Introduction

 The term Metaverse comes from the Greek prefix meta (meaning “between” or “beyond” and verse (“universe”). In his 1992 novel Snow Crash, science fiction author Neal Stephenson was the first to use this term [1].

The concept of the Metaverse refers to the possibility of transcending the physical world’s limitations and accessing a new virtual reality (VR) that exceeds geographical and biological boundaries.

Nowadays, Metaverse refers to a virtual reality existing beyond reality. It’s a digitized earth as a new world expressed through digital media such as smartphones and the internet [2].

After the metaverse concept appeared, extensive efforts and research were carried out to make it a reality, and the Metaverse roadmap [3] was announced in 2006.

In 2021, Go et al. defined the Metaverse as a 3D-based virtual where avatars representing real-world users perform daily activities [4]. These avatars engage in social, economic, and cultural activities in the Metaverse, marking an interaction and integration between the physical world and virtual reality [5].

Over time, the boundaries between the physical world and the Metaverse have become increasingly blurred. Real-life events and life in Metaverse increasingly overlap across all sectors [6].

Industries such as tourism, social networking, entertainment, agriculture, and education were the first real-world sectors to find direct applications within the metaverse [7–10] .

Since 2021, the scientific medical community has started questioning the possible uses of Metaverse in different medical specialties. Conversely, nobody has yet examined the potential of Metaverse’s forensic and legal medicine aspect.

So, this study seeks to establish the current Metaverse’s diffusion, exploration, and application level and its anticipated future in different medical specialties. To this end, our first task was to conduct what can be described as the first stage of the research in which all literature describing attempts to implement the Metaverse in healthcare was collected. This correlates with their analysis concerning the degree of interest and the specialization scope of a potential application. The analysis suggests that most medical specialties seem to have taken an interest in the promising applications of Metaverse and how it can be used beneficially in the fields of medicine, education, and patient management. This phenomenon is important as it highlights the potential of the Metaverse as an all-encompassing platform for addressing a wide range of issues within the healthcare system.

As interest in Metaverse increases among different medical specialties, the same is not valid for forensic and legal medicine, which both have shown an early and limited interest in the opportunities offered by Metaverse. Only a few book chapters and conference presentations discussed topics on security, social, and crime risks in Metaverse [11–13].

So, the second objective of this study was to suggest new forensic uses of the Metaverse and determine its prospects for further development in this area. Therefore, this paper attempts to answer a certain research question related to a new phenomenon that has received very little attention and needs further development in the forensic community. While the paper critically approaches the subject matter, it covers the existing uses of the Metaverse in medicine, as well as the future potential of this concept and its relevance in forensic engineering.

Attention was paid to forensic anthropology, crime investigations, forensic ballistics, crime scenarios in the metaverse, solving cold cases, and more, for which they aren’t available in the forensic literature.

The underlying question is: are forensic and legal medicine the singular domain that remains inattentive to the present possibilities and foreseeable advancements in the context of the Metaverse?

## Materials and methods

This scoping review was conducted by identifying the research questions, identifying relevant studies, selecting the studies, charting the data, and collating, summarizing, and reporting the results. The review was carried out in accordance with and is reported in accordance with the PRISMA-ScR (Preferred Reporting Items for Systematic Reviews and Meta-Analyses extension for Scoping Reviews) guidelines reporting guidance [14].

### Search strategy

Systematic search queries of PubMed Central, Web of Science, Scopus, and Cochrane were used to identify references published or available online until August 2024. This search was done from August to October 2022.

The search strategy employed the following keywords: “metaverse [Title/abstract]” for PubMed; “TS=(‘metaverse’) AND (TS=(‘medic*’ OR ‘health*’))” for Web of Science; “TS=(‘metaverse’) AND (TS=(‘medic*’ OR ‘health*’))” for Scopus; and “metaverse” in the Title Abstract Keyword fields for Cochrane.

### Eligibility criteria

After the obtained records were imported onto Rayyan, an online platform designed for multiple reviewers to work on systematic reviews, titles, and abstracts, they were reviewed for eligibility [15]. Reviewers are kept blind to each other’s decisions and can mark records as “include,” “exclude,” or “maybe.” They can also mark exclusion reasons or add notes. This process determined which records would be brought forward for full-text review.

Accordingly, to meet the eligibility criteria, the included papers had to be (1) original articles, (2) book chapters, (3) reviews, (4) systematic reviews, (5) editorials, and (6) articles in English.

Instead, the exclusion criteria were: (1) conference communications, (2) articles that only briefly discussed the Metaverse and medicine without providing in-depth analysis, and (3) articles not in English.

We chose to include nearly all types of publications in our review because this topic is uncharted, and we believed exploring a broad range of sources was essential. Nonetheless, we excluded conference communications due to their lack of context and clarity regarding the analysis scope. Our decision to select only English articles was based solely on the convenience of understanding the language without relying on a translator. Lastly, we omitted articles that merely touched on the Metaverse and medicine without offering in-depth analysis, as these did not fulfill the review’s aim of providing a clear understanding of the Metaverse’s development in the medical field.

### Study selection

Study selection was done in two steps. First, after duplicates were removed, the titles and abstracts of all retrieved articles were screened for eligibility by two authors. Next, the full text of all remaining publications was checked for inclusion by the same authors. Disagreements on including or excluding publications were discussed until agreement was reached. The average percentage of agreement between authors was approximately 95%.

## Results

The review was conducted on 1,893 articles, and after eliminating duplicates, 1,200 articles relevant to the Metaverse in clinical and healthcare contexts were selected for review. A total of 755 articles were excluded, while the remaining 445 articles met the study’s inclusion criteria.

We summarized the data obtained from our literature search using the PRISMA flowchart (Table 1).

**Table 1:**
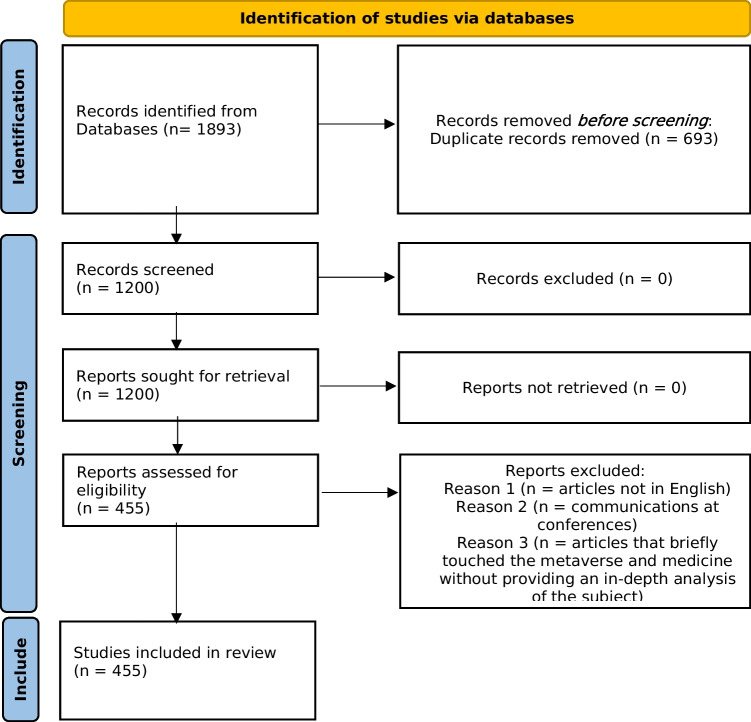
Summary of the data obtained from our literature search

We categorized the articles into three main groups (Table 2).

**Table 2:** Article on Metaverse categorized into 3 groups

Generic	Education	Medical Fields
202 (45.39%)	53 (11.91%)	190 (42.7%)

The first category, “Generic”, comprised 202 articles (45.39%) that discussed the potential interest of healthcare disciplines in general and theoretical terms in the Metaverse.

The second category, “Education”, comprised 53 articles (11.91%), representing a more academic or training-focused perspective. The authors of the articles did not specify a medical specialty but noted that the Metaverse is emerging as a significant tool for healthcare professional training. It allows for overcoming physical and geographical barriers and utilizing powerful virtual tools to offer advanced education, immersing students in clinical environments and allowing them to simulate medical procedures repeatedly.

The third category, “Medical Fields”, included 190 articles (42.7%), further subdivided into three groups: Clinical Specialties with 106 articles (23.82%); Surgical Specialties with 60 articles (13.48%); and Other Specialties (Supportive Medical Fields, Service-Oriented Medical Specialties) with 24 articles (5.39%).

The third category is summarized in Table 3.

**Table 3:** The distribution of Metaverse’s articles in Medical Fields

Clinical	Surgical	Supportive/ Service Oriented
106 (23.82%)	60 (13.48%)	24 (5.39%)

We find that 29 medical specialties report the applicability of the Metaverse within their field (Table 4), noting that forensic and legal medicine reported 0 articles, notwithstanding our research.

**Table 4:** Medical specialties that found interest in Metaverse

Field	Specialty	*N*. Of Articles	%
Clinical	Psychiatry/mental health	59	31.05%
Surgical	General Surgery	19	10.00%
Clinical	Neurology	11	5.79%
Clinical	Cardiology	10	5.26%
Surgical	Urology	10	5.26%
Clinical	Dermatology	9	4.74%
Surgical	Rehabilitation	9	4.74%
Surgical	Orthopedics	7	3.68%
Supportive	Anatomy	5	2.63%
Supportive	Anesthesiology	5	2.63%
Surgical	Neurosurgery	5	2.63%
Surgical	Dentistry	4	2.11%
Supportive	Diagnostic Imaging	4	2.11%
Clinical	Emergency Medicine	4	2.11%
Surgical	Gynecology	4	2.11%
Surgical	Plastic Surgery	4	2.11%
Clinical	Gastroenterology	3	1.58%
Clinical	Pneumology	3	1.58%
Clinical	Allergology	2	1.05%
Surgical	Breast Surgery	2	1.05%
Clinical	Oncology	2	1.05%
Surgical	Ophtalmology	2	1.05%
Clinical	Pediatrics	2	1.05%
Surgical	Cardiosurgery	1	0.53%
Supportive	Hygiene and Preventive Medicine	1	0.53%
Surgical	Pediatric Surgery	1	0.53%
Clinical	Rheumatology	1	0.53%
Surgical	Vascular Surgery	1	0.53%
Supportive	Forensic/Legal Medicine	0	0%

The medical specialties with the highest number of metaverse-related articles were Psychiatry (31.05%), General Surgery (10.00%), Neurology (5.79%), Cardiology, and Urology (5.26%).

The various medical specialties primarily identified five fields of application within the Metaverse (Table 5): “Future applicabilities” with 96 articles (50.52%); “Training” with 45 articles (23.68%); “Treatment” with 39 articles (20.53%); “Prediction/Prevention” with 7 articles (3.68%); and “Diagnosis” with 3 articles (1.58%).

**Table 5:** Fields of application within the Metaverse

Field of interest	*N*. Of Articles	%
Future applicabilities	96	50.52%
Training	45	23.68%
Treatment	39	20.53%
Prediction/prevent	7	3.68%
Diagnosis	3	1.58%

### Future applicabilities

Numerous medical specialties anticipated that a tool would be adopted shortly in daily clinical practice. Consequently, clinical practice may need to adapt and expand into the Metaverse.

A total of 23 medical specialties suggested such future applicabilities, with Psychiatry (34.37%) being the most prominent, mainly due to concerns related to mental health [16–18]. It was followed by General Surgery (11.46%) [19, 20], Cardiology (8.33%) [21, 22], Dermatology (7.29%) [23, 24], and Urology (6.25%) [25, 26].

All medical specialties proposing future applicabilities are summarized in Table 6.

**Table 6:** Metaverse’s “future applicabilities” articles in different medical specialties

Specialty	*N*. Of Articles	%
Psychiatry/mental health	33	34.37%
General Surgery	11	11.46%
Cardiology	8	8.33%
Dermatology	7	7.29%
Urology	6	6.25%
Neurology	5	5.21%
Anesthesiology	4	4.17%
Gynecology	3	3.13%
Allergology	2	2.08%
Dentistry	2	2.08%
Diagnostic Imaging	2	2.08%
Orthopedics	2	2.08%
Anatomy	1	1.04%
Emergency Medicine	1	1.04%
Gastroenterology	1	1.04%
Neurosurgery	1	1.04%
Oncology	1	1.04%
Ophthalmology	1	1.04%
Pediatrics	1	1.04%
Plastic Surgery	1	1.04%
Pneumology	1	1.04%
Rehabilitation	1	1.04%
Rheumatology	1	1.04%

### Training

A total of 21 specialties have indicated that the Metaverse is already a valuable tool for specialized training through simulation [27–29]. The specialties most interested in training within the Metaverse are General Surgery (17.78%) [30, 31], Orthopedics (8.89%) [32, 33], Neurosurgery (8.89%) [34, 35], and Anatomy (8.89%) [36, 37].

Some surgical branches have suggested performing practical simulations, such as conducting surgical procedures on avatars in the metaverse [30]. Others foresee the potential to simulate clinical conditions and pathologies using avatars, enhancing training by resolving each case [38]. Still, others envision the opportunity to study individual organs and perform technical exercises on specific systems [39].

Table 7 summarizes all the medical specialties showing an active interest in training within the Metaverse.

**Table 7:** Metaverse’s “training” articles in different medical specialties

Specialty	*N*. Of Articles	%
General Surgery	8	17.78%
Anatomy	4	8,89%
Neurosurgery	4	8.89%
Orthopedics	4	8.89%
Emergency Medicine	3	6.67%
Plastic Surgery	3	6.67%
Psichiatry/Mental Health	3	6.67%
Dentistry	2	4.44%
Diagnostic Imaging	2	4.44%
Urology	2	4.44%
Anesthesiology	1	2.22%
Breast Surgery	1	2.22%
Cardiology	1	2.22%
Cardiosurgery	1	2.22%
Gynecology	1	2.22%
Neurology	1	2.22%
Pneumology	1	2.22%
Ophtalmology	1	2.22%
Pediatric Surgery	1	2.22%
Vascular Surgery	1	2.22%

### Treatment

A total of 11 specialties have identified the Metaverse as a helpful tool for treatment purposes. The specialties showing the most significant interest in this area are Psychiatry (51.28%), Rehabilitation (20.51%), Neurology (5.13%), and Urology (5.13%).

Rehabilitation programs were suggested to be designed over the Metaverse with virtual coaches present in persistent and immersive virtual reality. These programs provide sophisticated results, instant modifications of their actions dependently on the user’s psychophysiological state, and the advantage of providing continuous emotional interactions within life scenarios [40, 41].

On the other hand, surgical specialties have mainly focused on the possibility of greater accuracy in surgical procedures, such as tumor removals, which have minimal errors compared to the real world [42].

All medical specialties that identified potential treatment applications within the Metaverse are summarized in Table 8.

**Table 8:** Metavers’s “treatment” articles in different medical specialties

Specialty	*N*. Of Articles	%
Psichiatry/Mental Health	20	51.28%
Rehabilitation	8	20.51%
Gastroenterology	3	7.69%
Neurology	2	5.13%
Urology	2	5.13%
Breast Surgery	1	2.56%
Cardiology	1	2.56%
Dermatology	1	2.56%
Pediatrics	1	2.56%
Pneumology	1	2.56%
Oncology	1	2.56%

### Prediction/Prevention

Four specialties have highlighted the role of the Metaverse in predictive and preventive medical applications. The specialties focusing on this aspect of medical practice are Psychiatry (42.86%), Neurology (28.57%), Orthopedics (14.29%), and Public Health (14.29%).

Studies in this field suggest that the Metaverse could serve as a space to promote health education, with a more significant impact on populations than what could be achieved through real-world tools [43]. Moreover, the Metaverse could eliminate geographical and economic barriers, facilitating, for instance, collaborations with humanitarian organizations by enabling countries with sufficient vaccines to help low-income nations with similar vaccination needs [44].

Table 9 summarizes all the medical specialties showing an active interest in prediction/prevention within the Metaverse.

**Table 9:** Metaverse’s “prediction/prevention” articles in different medical specialties

Specialty	*N*. Of Articles	%
Psychiatry/Mental Health	3	42.86%
Neurology	2	28.57%
Hygiene and Preventive Medicine	1	14.29%
Orthopedics	1	14.29%

### Diagnosis

Only three specialties have identified diagnostic utility within the Metaverse: Neurology (33.3%), Dermatology (33.3%), and Gastroenterology (33.3%).

These studies considered the applicability of diagnostic tests and models within the Metaverse, providing a more welcoming virtual environment than traditional healthcare settings. The ability to simulate home visits for patients requiring hospitalization for severe illnesses was also explored [45]. Additionally, using “smart glasses” during endoscopic procedures, allowing for a more direct and comprehensive evaluation of lesions compared to images produced by the endoscope, was proposed [46].

 Table 10 outlines the findings in this area.

**Table 10:** Metaverse’s “diagnosis” articles in different medical specialties

Specialty	*N*. Of Articles	%
Dermatology	1	33.33%
Gastroenterology	1	33.33%
Neurology	1	33.33%

## Discussion

The review identifies that nearly all medical specialties have shown direct interest in or identified potential future applications for the Metaverse.

The Metaverse links to experience in immersive virtual reality to customize new digital environments for practitioners and patients.

In particular, the area of medical specializations anticipated the ability to simulate clinical settings in a “risk-free” environment, allowing healthcare providers to assess and experiment with various behaviors and observe the consequences of their actions. Tutors could supervise these simulations, and specialists could be anywhere in the world [27]. The benefits, however, are not limited to healthcare providers. Patients with psychiatric and neurological diseases could also benefit from personalized diagnostic and therapeutic journeys in virtual environments tailored to their needs, often including disruptions in reality [47].

Similarly, surgical specialties could benefit from simulations of real, past, or hypothetical highly complex surgical scenarios on virtual avatars. These would be training exercises to improve operators’ skill, timing, and precision [19].

The other specialties (supportive medical fields, service-oriented medical specialties), being inherently supportive, could be integrated into the training protocols of clinical and surgical specialties, offering collaborative multidisciplinary learning platforms. These specialties could also be used to promote preventive health guidelines to a broader audience [44].

Forensic and legal medicine have yet to consider the Metaverse’s future applications or the contributions it could make to their disciplines.

Legal medicine has greater relevance to civil and tort law, impacting patient care, whereas forensic medicine relates to criminal law and damage to, or by, patients [48].

The lack of articles regarding Metaverse in forensic and legal medicine raises the question of its potential availability and applicability.

According to the other specialties’ perspectives, autopsy practice can be simulated in the virtual world, enabling forensic pathologists to conduct many autopsies even in unusual scenarios. Hence, Metaverse will support the training of young forensic pathologists.

Another feasibility could also be to create and simulate a crime investigation due to the availability to gather evidence and capture biological traces for establishing the cause and manner of death in a virtual setting.

As in other medical specialties, forensic doctors could also practice forensic cases, reconstructing and resolving them virtually.

These opportunities would be precious since forensic cases are epidemiologically rare compared to other medical disciplines.

Consider the Metaverse’s potential for creating virtual ballistics tests, complex homicides, mass disasters, personal injuries, and traffic accidents. Within the Metaverse, the forensic pathologist could return to such instances as often as possible, with absolute power over the limits of time and space.

Some of these reconstructions could even be used in real cold cases from the past, changing the verdicts in cases made on such occasions in the light of new possibilities provided by this new technology.

Numerous experts in the real world will have access to crime scenes reproduced in the Metaverse, and different hypotheses can be compared.

Another critical new frontier could involve assessing the biological harm inflicted on humans due to crimes committed between avatars in the Metaverse. If we can concretely use the Metaverse to solve crimes perpetrated in the real world, this is a critical issue to consider; perhaps in the future, some forensic doctors may be called upon to solve crimes perpetrated directly in the Metaverse. Accordingly, the Interpol perspective on Metaverse depicts virtual crime scenarios that include homicides, assault, or non-consensual and illicit offenses of sexual nature towards their avatars [49].

Some articles conduct an in-depth analysis of the Metaverse from the perspective of electronic data forensics, examining its development, security risks, and crime potential. The criminal risks of the Metaverse mainly include endangering national security, citizens’ personal information and privacy, online gambling, pornography, money laundering, and fraud [11]. Some studies also explore forensic procedures proposing smart bracelet forensics and tools developed for metaverse digital forensics, addressing gaps in current investigative frameworks [12, 13].

Millions of dollars are spent daily to buy avatars and properties within the Metaverse. It is well-known that humans tend to personify their avatars, and the psychological suffering and potential pathology experienced by one’s avatar could, in turn, affect the real person.

We must all imagine the future in light of exponential technological development, increasingly bringing the Metaverse closer to our reality.

Shortly, it will be possible to create metaverse experiences that immerse human beings so thoroughly that they generate complete sensory engagement and biofeedback.

Forensic doctors could simulate real events in the Metaverse with such precision that they would no longer be mere virtual simulations but a different and tangible reality [50].

Several authors have developed systems that support scene work and aim to support subsequent analysis in the lab. Gee et al. (2010) developed a mobile AR system that is based on a backpack computer, a global positioning system (GPS) for tracking, and a camera and a touch screen. This system allows a forensic investigator to create a digital incident map of the scenery through simultaneous localization and mapping (SLAM) and add annotations. When other investigators return to the crime scene, they can use these annotations to quickly orient themselves and obtain a better overview of the scenery. Multiple persons can also use the system to improve collaboration [51].

The future will mix the worlds that exist separately: the material one and the Metaverse, which will eventually come together into one dimension.

Today, other technological tools, such as artificial intelligence, are likewise developing uncontrollably. AI could be used to estimate the post-mortem interval using microbial data [52, 53]. The impact that incorporating these new technologies will have on our planet and lives is beyond our wildest expectations.

However, using the Metaverse in forensic and legal medicine introduces various ethical challenges that require careful consideration. One major issue is ensuring the accuracy and reliability of virtual evidence and simulations. For example, when crime scenes are reconstructed in a virtual environment, it is crucial to replicate them as precisely as possible to avoid errors that could impact legal decisions. Virtual evidence is vulnerable to tampering, whether deliberate or accidental, emphasizing the critical need for robust safeguards and reliable security measures to maintain its integrity. Additionally, the highly immersive nature of the Metaverse can make it difficult to separate reality from simulation, potentially affecting the judgment of jurors or trainees who interact with these tools.

Virtual crimes, like those targeting avatars, such as hacking an avatar to impersonate its owner or engaging in virtual harassment, add another layer of complexity.

add another layer of complexity.

Defining what is harmful in a virtual environment and figuring out how to hold individuals accountable for their actions pose significant challenges. For instance, if someone’s avatar experiences a harmful act, determining whether this translates to real-world consequences and how to address such situations legally can be difficult. Moreover, the collection and use of digital data in virtual forensic investigations raise concerns about privacy, data ownership, and security. Proper rules and practices are needed to manage these issues responsibly.

To address these challenges, it would be prudent to establish ethical committees to oversee the use of virtual technologies in forensic contexts and develop standardized guidelines for their application. Legal systems could consider exploring regulations to govern the admissibility and integrity of virtual evidence in courts. Collaborative efforts between technologists, lawyers, and ethicists should aim to create robust frameworks that enhance privacy, security, and fairness. Additionally, fostering cross-disciplinary dialogue and prioritizing creating comprehensive policies would be essential steps toward addressing these complex issues effectively.

In conclusion, integrating the Metaverse into forensic and legal medicine requires clear ethical guidelines and technical standards. These should focus on ensuring the accuracy and security of virtual evidence, protecting data privacy, addressing biases in AI tools, and making advanced technologies accessible to all. By tackling these challenges, researchers and professionals can maximize the benefits of the Metaverse while staying true to fundamental ethical principles.

## Conclusion

The Metaverse has gone from a theoretical standpoint to being rendered in practice across many industries, and healthcare is just one of the major sectors. The review highlights how all medical specialties explore and adopt metaverse-based solutions in education, training, treatment, and predictive and preventive care. However, forensic pathology and legal medicine need to catch up in these discussions, raising significant questions about whether these fields are underestimating the potential of Metevarse.

Since ample evidence suggests the necessity of the Metaverse in social and surgical domains, the field of forensic sciences and legal medicine would also extensively utilize such 3D interactive systems. Among these could be valuable virtual training in autopsy performance, crime scene reconstruction, and more applied aspects of forensics where the number of cases is limited. Also, historical criminalistics can be revisited from the perspective of the Metaverse, and its potential for evaluating psychological and biological harm in and out of virtual space is also worth considering in legal scenarios.

With the gradual blurring of the boundary between the physical world and the Metaverse, adopting metaverse technologies in forensic science and legal medicine is no longer an option but a viable target. The prospect for the future is one in which one can take advantage of the Metaverse, in which one is completely cut off from the real world but uses real-life medicine and forensic science practices.

Nevertheless, achieving the successful integration of the Metaverse into forensic and legal medicine hinges on developing clear ethical guidelines and technical standards, ensuring the accuracy and security of virtual evidence, safeguarding data privacy, and addressing the eventual application of AI systems and their possible biases.

In this new technological frontier, it is time for forensic sciences to evolve and innovate with novel solutions that define the future of forensic science.

## Data Availability

N/A.
